# Content Validity of the Comprehensive ICF Core Set for Children with Cerebral Palsy Aged 0-6 Years: Iranian Occupational Therapists Perspective

**Published:** 2018

**Authors:** Parvin RAJI, Afsoon HASSANI MEHRABAN, Faranak ALIABADI, Maryam AHMADI, Veronica SCHIARITI

**Affiliations:** 1 Department of Occupational Therapy, School of Rehabilitation Sciences, Iran University of Medical Sciences, Tehran, Iran; 2 Department of Occupational Therapy, Rehabilitation Research Center School of Rehabilitation Sciences ,Iran University of Medical Sciences ,Tehran,Iran; 3 School of Rehabilitation Sciences, Rehabilitation Research Center, Iran University of Medical Sciences, Tehran, Iran; 4 Department of Health Information Management, School of Health Management and Information Sciences, Iran University of Medical Sciences, Tehran, Iran; 5 Department of Pediatrics, University of British Columbia, Vancouver, Canada; 6 Division of Medical Sciences, University of Victoria, British Columbia, Canada

**Keywords:** Cerebral palsy, Children, Evaluation, ICF, Occupational therapy, Validation

## Abstract

**Objectives:**

Comprehensive ICF Core Set of cerebral palsy (CP) includes a set of functions of children with CP has been created recently. This study determined the content validity of this version based on Iranian Occupational Therapists’ perspectives to explore whether the ICF Core Sets for CP include the areas of function of CP in Occupational Therapy practice.

**Materials & Methods:**

This qualitative study conducted from Feb 2015 to Apr 2016 in Tehran, Iran. Experts were the academic staffs selected through convenience sampling. Content validity of comprehensive ICF-Core Set of CP with 135 ICF categories was done by them. Delphi survey was used for generating consensus on the final version. Participants were 50 clinical Occupational Therapists invited via email from across Iran. An agreement of 75% was considered as the cut-off for inclusion of each code-category.

**Results:**

About 60% of the code–categories of comprehensive version of ICF Core Set of CP were approved by Occupational Therapists. In the final version, 82 code-categories were listed that included 21 code-categories for Body Functions, 40 for Activity/Participation, and 21 for Environmental Factors.

**Conclusion:**

The validity of the Iranian ICF Core Set for children with CP aged 0–6 yr was supported by Iranian Occupational Therapists. It could be the basis for evaluation of this population in Occupational Therapy.

## Introduction

Cerebral palsy (CP) refers to a group of movement disorders that is permanent and non-progressive damage to the developing brain that causes functional limitation in patients (1, 2). This is the most common cause of physical disability in early childhood, with the prevalence of 2-3.5 cases per 1000 live births in developed and developing countries (3). The prevalence of CP is 2-2.6 in 1000 live births in Iran (4). 

CP is often associated with disorders such as visual and hearing dysfunction, perceptual, cognitive, communication and behavioral disorders, epilepsy and musculoskeletal problems. These problems eventually change their quality of life and impose too much stress on caregivers. There are many problems in these children that varied and difficult to check (5, 6).

Today, using the classification system is self-evident and necessary especially in medical sciences (7). One of the classification systems, the international classification of functioning and disability and health (ICF), is currently in clinical research and will be using it a lot (8). It provides a holistic framework that describes relevant functional information about individuals with a health condition. Professionals from the health, education and social sectors are encouraged to apply the ICF framework in day-to-day practice (9). Owing to the multidimensional approach proposed by the ICF, occupational therapists are increasingly adopting the ICF framework and coding system in their practices (10). This category-based system and its pediatric version, the ICF for children and youth (ICF-CY), facilitates the observation and documentation of functioning of clients (10, 11) and provides an effective monitoring and evaluation tool for the assessment of intervention results (12, 13). Knowledge of ICF helped multiple disciplines to comprehensively evaluate individuals with CP (8, 14). Teaching physiotherapy students to assess patients based on the ICF provided a conceptual framework that guided their questions and allowed them to organize and integrate information using various components of ICF (15). 

To facilitate the application of the ICF and ICF-CY, ICF Core Sets have been created. These are shortlists derived from the ICF to facilitate the application in clinical practice for specific health conditions. There are two categories, brief ICF Core Sets that are minimal standards for reporting functioning and for collecting data in epidemiological studies, and the comprehensive ICF Core Sets that are standards for interdisciplinary and comprehensive assessments (16).

Several ICF Core Sets have been developed for various conditions. The ICF Core Sets for children and youth was developed with CP (CP) (17-22). CP ICF Core Set is a shortlist of ICF-CY containing specific functions for this population. ICF Core Set of CP has five versions: comprehensive for individuals 0–18 yr with 135 ICF categories; a common brief for 0–18 yr with 25 categories, a brief for 0–6 yr with 31 categories; a brief for 6–14 yr with 35 categories; and a brief for 14–18 yr with 37 categories (21). Use of the Comprehensive ICF Core Set of CP guides Occupational Therapists in their clinical practice (10).

In our study, the authors needed a framework to allow for better perception of function in this population and to facilitate the evaluation process for Occupational Therapists assessing children aged 0–6 yr with CP. We selected the comprehensive ICF Core Set of individuals' aged 0–18 yr with CP. 

The integration does not exist among specialists in identifying the problems and needs of this population that causes poor assessment and inconsistency among the experts (8). Physiotherapists and Occupational Therapists have key roles in motor interventions for children with CP (23). Children aged 0–6 yr with CP constitutes the largest referral population to Occupational Therapy centers in Iran. The progress and response to intervention in children below 6 yr of age are difficult to track, and currently, there is no integrated program available for evaluation of this population. On the other hand, 0–6 yr is an age range used in the ICF Core Set brief version (21) and rehabilitation during this age period is more effective than at other ages.

Despite efforts have been made for organizing of evaluation of CP in occupational therapy clinics; there is no standard form or checklists for evaluation of these children. This causes confusion among students and even occupational therapists in evaluation, decision-making, goal setting and intervention of CP. If there is a valid system in evaluation process, acceptability and usage of that system will increase. Aim of this preliminary study was identification of function of these children based on occupational therapy perspective on the ICF-Core Set of CP. 

The present study was designed to validate CP ICF Core Set from Occupational Therapy perspective to monitor children aged 0 to 6 yr with CP. Before using ICF Core Sets for children with CP, several content validity studies should be performed (24). Because under-representation of experts from different countries may have led to cultural bias. Therefore, investigating the effect of different cultural, social, educational, economical and health system on ICF-Core Sets will help using better across countries (22, 24). Some authors studies have considered the validity of ICF Core Sets for other diseases from the perspective of Occupational Therapists, for example, rheumatoid arthritis (25), stroke (12), spinal cord injury (26), and multiple sclerosis (27). In fact, after doing this preliminary research we can organize current tools or prepare tools based on the validated ICF Core Sets (15).

## Materials & Methods

**Participants**

Participants were two groups. One group of 12 expert Occupational Therapists who were academic and experienced in the evaluation and treatment of children with CP. All were considered an expert in using ICF and had at least 10 yr work experience in rehabilitation of CP children, had postgraduate Occupational Therapy degrees (at least an MSc), and publication of at least one article about CP or the ICF. They were selected by convenience sampling. This type of sampling is commonly used in qualitative studies and there is no agreement on the number of participants in various studies (28). These 12 Occupational Therapists participated in the panels and content validity study.

Participants in the second group (n=50) were clinical and experienced Occupational Therapists experienced in the evaluation and treatment of cerebral palsy, had at least 5 yr experience working with CP children in outpatients/hospital centers, and at least a bachelor's degree in Occupational Therapy. They were selected from across the country. Since in the Iranian Occupational Therapy Association there was address, contact number and e-mail of occupational therapists, the framework of the study were sent them via email. Of the respondents, 50 met inclusion criteria. The number of participants in Delphi studies varies from 15 to 60 people (29). These 50 Occupational Therapists participated in Delphi survey.

**Procedure**

The study was approved by the IUMS Ethics Committee (number 93/د/105/5472). It was a qualitative study using expert panels and Delphi survey in four phases from Feb 2015 to Apr 2016 in Tehran at the Iran University of Medical Sciences (IUMS). 

 1. The first phase centered on framework selection and preparation of four questions about the chosen framework in the expert panel. As the brief ICF Core Set for children aged 0–6 yr does not represent all the functions of this age range (21, 24) and is used for brief assessment (21), it was decided to use the comprehensive ICF Core Set of CP as the study framework. The translation code-categories were selected from main ICF book in Persian (30). A question was posed at the beginning of each component of the comprehensive ICF Core Set. For example, for Body Structures, the question was: Is assessment of these categories necessary for children with CP aged 0–6 yr in Occupational Therapy outpatient centers? This question was repeated for the other three components (Body Functions, Activity and Participation, and Environmental Factors). Experts viewed the framework and answered the questions. In cases, which an agreement reached over 75 percent, code-category approved.

2. In the second phase, content validity was qualitatively described. To date, several of the ICF Core Sets have been evaluated. To our knowledge, this study is the first to include validation following the development of the CP ICF Core Sets. For qualitative validation that consisted of two expert panels, ICF and its definitions and ICF Core Sets of CP were introduced to the experts, together with the aim of the study. In the first expert panel, from the comprehensive version of ICF Core Set (0–18 yr) in Persian, code-categories suitable, and related to children aged 0–6 yr with CP were selected by the experts. An agreement of 75% was considered as the cut-off for inclusion of each code-category, and 50%-75% for discussion in the next expert panel (31). Agreement of 75% for each code-category means that there is agreement among 75% of experts about that code-category. In the second expert panel, code-categories with 50%-75% agreement were reexamined. It took two hours for per panel.

3. In the third phase, content validity was quantitatively described by determination of the Content Validity Ratio (CVR) and Content Validity Index (CVI). For quantitative validation, the final version of two previous expert panels was sent them requesting a response within a 2-wk period via email. After completion, ultimately consensus was reached about the results.

4. In the fourth phase, the method was Delphi. It is a multi-stage method of qualitative research in which experts comment, in relation to the particular topic or question. Usually, each round is based on the results of the previous round. Usually, Delphi survey performed in two or three rounds to achieve consistency in results (25, 29). The consensus was achieved with two rounds and a mini Delphi. Delphi method was used on the basis of Fowles stages (32). In the first round, validated ICF Core Set of CP was sent to occupational therapists across the country via email. At the beginning of the email explained about the definition of ICF and ICF Core Set, Delphi method and purpose of the project and requested demographic information from eligible participants. In front of each code-category, the question was: Is assessment of these categories necessary for children with CP aged 0–6 yr in Occupational Therapy outpatient centers? The code-categories were accepted by over 75% agreement, 50% to 75% agreement were postponed to the next round, and lower than 50% agreement were removed (31) because the most common definition for consensus is 75% (31). After two weeks, a reminder was sent to respondents. In the second round, all items with 50%-75% agreement were sent again to respondents. Usually, Delphi rounds continue until a consensus is reached. A mini Delphi method was used for a few changes in the definitions of some code-categories. Finally, clinical occupational therapists made a few changes and prepared a final set. Mini Delphi is a reliable procedure replaced a Delphi round and will include more detailed comments (33). 

**Data analysis**

Content validity was qualitatively evaluated by two expert panels and quantitatively by determination of CVR and CVI with final decision-making. Content validity expressed as a quantitative value denoting the degree of which the instrument covers the content. For validation, CVR and CVI were calculated. CVR is a measure (34). Experts respond to questions using a forced choice paradigm by indicating ‘essential’, ‘useful but not essential’, and ‘not essential.’ In this formula, n_E_ is the number of essential responses, and N is the number of experts participating. 

$\mathrm{CVR}=\frac{n_{E}-\frac{N}{2}}{\frac{N}{2}}$

The CVI uses a four-point Likert scale to rate each code-category based on ‘simplicity’, ‘relevancy’, or ‘specificity’, and ‘clarity’. Every code-category was calculated in terms of these three features. CVI was computed as the number of experts giving a rating of either 3 or 4 for each indicator, divided by the number of experts; that is, the proportion in agreement about relevancy, simplicity, or clarity (35-37). The score for suitable code-categories was greater than 0.79; for questionable categories was a value between 0.70–0.79, and for unacceptable code-categories, it was less than 0.70 (35-37). This rule adhered to the panel and Delphi stages.

## Results

**Participants**

Table 1 shows demographic and professional characteristics of the 12 expert Occupational Therapists and 50 clinical Occupational Therapists. In the first round, 50 (100%) Occupational Therapists (30 males and 20 females) participated, in round 2, 30 (60%) (17 female, 13 male) and in the mini Delphi 10 (20%) (5 women and 5 men). 

**Table 1:** Demographic and professional characteristics of the expert or academic (N=12) and clinical occupational therapists (N=50) participating in the study

**Participants**	Academic Occupational Therapist	Clinical Occupational Therapist
**Variable**		
**Age (year)**	Average 40±6 yr	Average 34±6 yr
**Sex**	7 male, 5 female	20 male, 30 female
**Experience in field of CP(year)**	Average 15±4 yr	Average 9±4 yr
**Education**	6 PhD, 5 currently registered for PhD, 1 MSc	2 PhD, 7 currently registered for PhD, 23 MSc, 5 currently registered for MSc, 13 BS
**Main work setting**	University and outpatient rehabilitation centers	Outpatient rehabilitation centers

**Content validity results**

The first expert panel considered all 135 categories from the selected framework. Of these, 73 code-categories were accepted (agreement greater than 75%), 25 code-categories were omitted (agreement less than 50%), and 37 code-categories were borderline (agreement 50%–75%). Thirty-one code-categories related to the brief version of children aged 0–6 yr were accepted, except for mental function of language and hearing functions. Experts suggested that five code-categories be added, including memory, vestibular, touch and motor reflex functions, and undertaking a single task. In the second expert panel, a decision was made regarding the code-categories with 50% to 75% agreement (38, 39). The result of this stage was 22 accepted code-categories, and 22 judged as ineligible. 

Following two expert panels, 95 accepted code-categories were obtained (73 from the first panel and 22 from the second). The 95 code-categories included four for Body Structure, 27 for Body Functions, 40 for Activity and Participation, and 24 for Environmental Factors. Some of the code-categories in the brief ICF Core Set were not included in our output (seeing and hearing functions, and mental functions of language). 

For quantitative validation, a framework was prepared with 95 code-categories for children (0–6 yr) and ten code-categories were omitted as they had CVRs of less than 0.56 (based on an appraisal of 12 experts) and CVIs of lower than 0.79. In addition, 25 code-categories were modified. In total, 84 code-categories were accepted with 21 code-categories for Body Function, 40 for Activity and Participation, and 23 for Environmental Factors (Table 2). 

**Table 2:** ICF Core Set code-categories accepted as suitable by the expert panels, based on CVR and CVI results

	**Component**	**Accepted code-categories**	**Comments**		**Component**	**Accepted code-categories**	**Comments**
1	Body Function	B117 intellectual functions		43	Activity/Participation	D435 moving objects with lower extremities	
2		B1301 motivation & b152 emotional functions	Merged	44		D440 fine hand use	
3		B140 attention functions		45		D445 hand and arm use	
4		B144 memory functions*		46		D450 walking	
5		B156 perceptual functions		47		D455 moving around	Changed to moving with every style except walking
6		B163 basic cognitive functions	Changed to fundamental cognitive functions	48		D460 moving around in different locations	Changed to walking and moving with every style in different locations
7		B235 vestibular functions*		49		D465 moving around using equipment	Changed to moving around using therapeutic and nontherapeutic equipment
8		B260 proprioceptive function		50		D510 washing oneself	
9		B265 touch functions*		51		D520 caring for body parts	
10		B280 sensation of pain		52		D530 toileting	
11		B510 ingestion functions		53		D540 dressing	
12		B710 mobility of joint functions		54		D550 eating	
13		B715 stability of joint functions		55		D560 drinking	
14		B730 muscle power functions		56		D710 basic interpersonal interactions	
15		B735 muscle tone functions		57		D720 complex interpersonal interactions	
16		B740 muscle endurance functions		58		D760 family relationships	
17		B7508 motor reflex functions*		59		D815 preschool education	
18		B755 involuntary movement reaction functions		60		D880 engagement in play	
19		B760 control of voluntary movement functions		61		D920 recreation and leisure	
20		B765 involuntary movement functions	Changed to involuntary abnormal movement functions	62	Environmental factors	E110 products or substances for personal consumption	Changed to drinking, food, and drugs
21		B770 gait pattern functions		63		E115 products and technology for personal use in daily living	Changed to equipment and technology for personal use in daily living
22	Activity/Participation	D110 watching	Changed to purposeful watching	64		E120 products and technology for personal indoor and outdoor mobility and transportation	Changed to equipment and technology for personal indoor and outdoor mobility and transportation
23		D115 listening	Changed to purposeful listening	65		E125 products and technology for communication	Changed to equipment and technology for communication
24		D120 another purposeful sensing	Changed to purposeful use of other senses	66		E130 products and technology for education	Changed to equipment and technology for education
25		D130 copying		67		E140 products and technology for culture, recreation, and sport	Changed to equipment and technology for culture, recreation, and sport
26		D131 learning through actions with objects		68		E150 design, construction and building products and technology of buildings for public use	Changed to design, construction and building equipment and technology of buildings for public use
27		D133 acquiring language		69		E155 design, construction and building products and technology of buildings for private use	Changed to design, construction and building equipment and technology of buildings for private use
28		D137 acquiring concepts		70		E310 immediate family	Changed in relation to immediate family and their support
29		D155 acquiring skills		71		E315 extended family	Changed in relation to extended family and their support
30		D160 focusing attention		72		E320 friends	Changed in relation to friends and their support
31		D175 solving problems		73		E325 acquaintances, peers, colleagues, neighbors and community members	
32		D177 making decisions		74		E340 personal care providers and personal assistants	Changed to personal care providers and caregivers
33		D210 undertaking a single task*		75		E355 health professionals	
34		D230 carrying out daily routine		76		E410 individual attitudes of immediate family members	
35		D250 managing one’s behavior		77		E415 individual attitudes of extended family members	
36		D310 communicating with-receiving-spoken messages	Changed to receiving and communicating with spoken messages	78		E420 individual attitudes of friends	
37		D335 producing non-verbal messages		79		E425 individual attitudes of acquaintances, peers, colleagues, neighbors and community members	
38		D350 conversation		80		E440 individual attitudes of personal care providers and personal assistants	Changed to individual attitudes of personal care providers and caregivers
39		D410 changing basic body position		81		E450 individual attitudes of health professionals	
40		D415 maintaining a body position		82		E460 societal attitudes	
41		D420 transferring oneself		83		E465 social norms, practices and ideologies	
42		D430 lifting and carrying objects		84		E570 social security services, systems and policies	

* Added from the first expert panel

**Accepted (included) code-categories**

Twenty-one code-categories for Body Functions, 40 code-categories for Activity and Participation, and 23 code-categories for Environmental Factors were included, giving 84 code-categories. Of the 84 code-categories, two code-categories are at the third level (b7508 motor reflex functions and b1301 motivation) and the remainder located at the second level. Eighty-seven percent of code-categories in the brief version for 0–6 yr (27 code-categories) and 73% of those from the comprehensive (0–18 yr) (79 code-categories) are in the framework. 

**Excluded code-categories**

There were two reasons for exclusion: age group of children and Occupational Therapists responsibilities. We used the comprehensive version of ICF Core Set of CP for children aged 0–18 yr of age, which meant that some code-categories were excluded such as d166 reading, d170 writing, d172 calculating, and d820 school education. Some were excluded because they fell beyond the scope of Occupational Therapists responsibility such as b530 weight maintenance functions and b620 urination functions and body Structure code-categories such as s320 structure of mouth, and s7700 bones. 

**Added code-categories**

According to expert’s comments, five code-categories were added including b144 memory functions, b235 vestibular functions, b265 touch functions, b7508 motor reflex functions, and d210 undertaking a single task, i.e. four code-categories related to Body Functions and one code-category (d210) related to Activity and Participation not covered by the Comprehensive ICF Core Set for CP. These ICF-CY code-categories are essential in Occupational Therapy evaluation and intervention with children aged 0–6 yr with CP.

**Modified code-categories**

Twenty-five code-categories were modified by the experts during the validation phases. Most of them were reworded to give a better sense of the concept or better understanding (Table 2). 

**Delphi rounds results**

In the first round, among 84 code-categories accepted by experts, 82 code-categories achieved greater than 75% agreement and two code-categories between 50% and 75% agreement. Interestingly, view of both groups [academic (expert) and clinical Occupational Therapists] except in two codes was the same. Only e315 and e415 did not achieve agreement greater than 75% (Table 3).

**Table 3:** Code-categories reached ≥75% agreement in the first round (N=50)

	**Code-categories**	**Round 1 n=50** **n(%) agreement**		**Code-categories**	**Round 1 n=50** **n(%) agreement**
1	b117 intellectual functions	50(100)	43	d435 moving objects with lower extremities	47(94)
2	b1301 motivation & b152 emotional functions	50(100)	44	d440 fine hand use	50(100)
3	b140 attention functions	50(100)	45	d445 hand and arm use	50(100)
4	b144 memory functions	48(96)	46	d450 walking	50(100)
5	b156 perceptual functions	48(96)	47	d455 moving with every style except walking	50(100)
6	b163 fundamental cognitive functions	47(94)	48	d460 walking and moving with every style in different locations	49(98)
7	b235 vestibular functions	50(100)	49	d465 moving around using therapeutic and nontherapeutic equipment	49(98)
8	b260 proprioceptive function	49(98)	50	d510 washing oneself	47(94)
9	b265 touch functions	49(98)	51	d520 caring for body parts	44(88)
10	b280 sensation of pain	49(98)	52	d530 toileting	48(96)
11	b510 ingestion functions	49(98)	53	d540 dressing	49(98)
12	b710 mobility of joint functions	50(100)	54	d550 eating	49(98)
13	b715 stability of joint functions	50(100)	55	d560 drinking	49(98)
14	b730 muscle power functions	49(98)	56	d710 basic interpersonal interactions	43(90)
15	b735 muscle tone functions	50(100)	57	d720 complex interpersonal interactions	41(82)
16	b740 muscle endurance functions	50(100)	58	d760 family relationships	42(84)
17	b7508 motor reflex functions	50(100)	59	d815 preschool education	46(92)
18	b755 involuntary movement reaction functions	49(98)	60	d880 engagement in play	49(98)
19	b760 control of voluntary movement functions	50(100)	61	d920 recreation and leisure	44(88)
20	b765 involuntary movement functions	49(98)	62	e110 drinking, food and drugs	46(92)
21	b770 gait pattern functions	49(98)	63	e115 to equipment and technology for personal use in daily living	49(98)
22	d110 purposeful watching	49(98)	64	e120 equipment and technology for personal indoor and outdoor mobility and transportation	49(98)
23	d115 purposeful listening	49(98)	65	e125 products and technology for communication	43(86)
24	d120 purposeful use of other senses	49(98)	66	e130 equipments and technology for education	47(94)
25	d130 copying	47(94)	67	e140 equipments and technology for culture, recreation and sport	46(92)
26	d131 learning through actions with objects	46(92)	68	e150 design, construction and building products and technology of buildings for public use	44(88)
27	d133 acquiring language	49(98)	69	e155 design, construction and building equipments and technology of buildings for private use	43(86)
28	d137 acquiring concepts	47(94)	70	e310 relation to immediate family and their support	45(90)
29	d155 acquiring skills	49(98)	**71**	**e315 relation to extended family and their support**	**34(68)**
30	d160 focusing attention	47(94)	72	e320 relation to friends and their support	44(88)
31	d175 solving problems	44(88)	73	e325 acquaintances, peers, colleagues, neighbors and community members	42(84)
32	d177 making decisions	42(84)	74	e340 personal care providers and personal assistants	43(86)
33	d210 undertaking a single task	39(78)	75	e355 health professionals	46(92)
34	d230 carrying out daily routine	46(92)	76	e410 individual attitudes of immediate family members	44(88)
35	d250 managing one’s behavior	41(82)	**77**	**e415 individual attitudes of extended family members**	**33(66)**
36	d310 receiving and communicating with spoken messages	44(88)	78	e420 individual attitudes of friends	40(80)
37	d335 producing non-verbal messages	46(92)	79	e425 individual attitudes of acquaintances, peers, colleagues, neighbors and community members	40(80)
38	d350 conversation	47(94)	80	e440 individual attitudes of personal care providers and caregivers	40(80)
39	d410 changing basic body position	50(100)	81	e450 individual attitudes of health professionals	41(82)
40	d415 maintaining a body position	50(100)	82	e460 societal attitudes	42(84)
41	d420 transferring oneself	50(100)	83	e465 social norms, practices and ideologies	49(78)
42	d430 lifting and carrying objects	48(96)	84	e570 social security services, systems, and policies	42(84)

In the second round, two code-categories - e315 (83%), e415 (76%)- achieved greater than 75% agreement by 30 Occupational Therapists for exclusion. Since consensus achieved after two rounds, 10 Occupational Therapists participated in a mini Delphi session for only the decision-making in the definitions of some code-categories that in previous rounds had been suggested.

**Figure 1:**
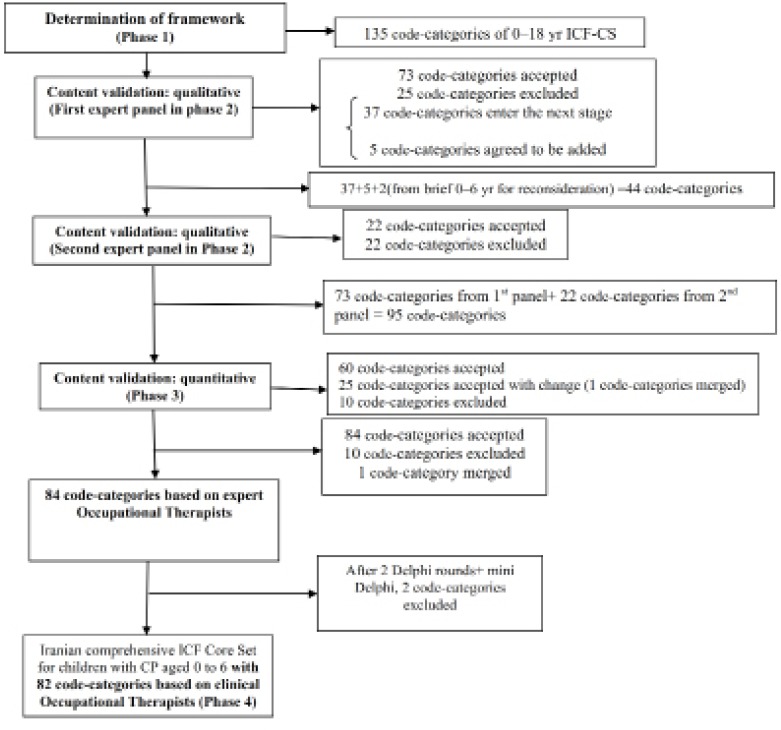
Overview of study to show how 82 code-categories were derived for the ICF Core Set for children with CP aged 0-6 yr

The way in which the code-categories were derived over the course of the study to yield 82 code-categories (Figure 1).

## Discussion

The aim of this study was to determine the validity of ICF-Core Set of CP based on Iranian Occupational Therapists’ perspectives for children 0 to 6 yr. Our study showed the high validity of comprehensive version of ICF Core Set.

ICF provides a framework for individual assessment (40). Our framework was designed with the same principles in mind but focusing specifically on what Occupational Therapists should know when assessing young children with CP. The validation process meant that some code-categories remained, others were excluded or modified and additional code-categories were added. In the final accepted version by expert panels, 84 code-categories, and after Delphi stages 82 code-categories were listed (Figure 1) that included 21 code-categories for Body Functions, 40 for Activity and Participation, and 21 for Environmental Factors. Therefore, 98% of the selected codes in both groups -academic and clinical occupational therapists- were similar. In Delphi, 2 code-categories of environmental factors "relation to extended family and their support "and" individual attitudes of extended family members" were excluded. Participants believed that "extended family" is not considered as facilitator or barrier for CP children. 

In a study with the lower percentage of agreement and adding more codes, have claimed validity of ICF Core Set, for example, 33% of the ICF Core set of Multiple Sclerosis (46 Codes from 138 Codes) and 19 codes were added (41).

The largest number of identified ICF code-categories belonged to the Activity and Participation component. These code-categories should be considered when assessing children, such as purposeful watching and listening, changing basic body position, fine hand use, eating, drinking, toileting, drinking, recreation, and leisure. Considering the 40 code-categories related to Activity and Participation, most were related to chapter 1 (Learning and applying knowledge) and chapter 4 (Mobility), each with 11 code-categories. There were also six code-categories related to chapter 5 (Self-care). These are representative of Occupational Therapists’ focus on these issues. Activity and Participation are considered key component for the CP children, in contrast to Body Function and Environmental Factors. This may reflect the fact that Activity and Participation component forms the central core of Occupational Therapy practice (25). Outcome measures in CP were reported, focusing on Body Functions, and Activity and Participation, with Environmental Factors, only partly considered (17). Andrade et al. identified codes related to CP assessment using ICF as an interdisciplinary framework in a retrospective study. As for our study, the majority of the codes identified by Occupational Therapists were related to Activity and Participation. Code-categories categorized by Occupational Therapists were different to those in this study. For example, ‘speaking’ was included in their survey but excluded in our study. The reason for this difference may have been that the scope of Occupational Therapy practice was not delineated in their investigation (42).

The number of Body Structure code-categories was very limited (17), more emphasis be placed on Activity and Participation as the current definition for CP mainly focuses on physical issues: CP describes a group of permanent disorders of the development of movement and posture, causing activity limitation, attributed to nonprogressive disturbances that occurred in the developing fetal or infant brain. “The motor disorders of CP are often accompanied by disturbances of sensation, perception, cognition, communication, and behavior, by epilepsy, and by secondary musculoskeletal problems” (1, 2). 

About 21 code-categories of body functions, most of them related to Chapter 7 (Neuromusculoskeletal and movement-related functions) and most codes in the 21 code-categories of environmental factors also were related to chapter 1 (Products and technology). Most attention in this area is paid to these matters by Occupational Therapists. Evaluation of Environmental Factors and Body Function are the responsibility of Occupational Therapists, who may carry out interventions in these areas (43). The aim was to identify CP functions for their evaluation based on ICF. In this study, children, parents and medical experts were asked to consider the problems of the person with CP. From 322 responses, 45% were related to chapter 4 (Mobility) and 45% related to Chapter 7 (Neuromusculoskeletal and movement-related functions) (44) that is compatible with our study. Mobility problems are a priority for most of the professionals associated with this group of people. Of course, children with CP stated greatest difficulties in mobility, self-care, and leisure, while caregivers mentioned the physical limitations and environmental factors are their main concern (11). Caregivers’ perspective is a little farther than the other two groups view. Fortunately, Occupational Therapists have the client-centered approach, in order to achieve the best results, therefore, they consider all three groups attitudes in the evaluation and intervention.

The protocol used for this study was novel and differed to those used in prior validation studies (12, 25-27). In other validation studies, ICF categories were derived after the Delphi process with open questions about disorders (12, 25-27), while in the current study, ICF categories were introduced to the Occupational Therapists at first because those code-categories were the framework of our study and our participants were not very familiar with them. Explanations of some categories in ICF Core Set of CP, especially concerning Environmental Factors, were unclear and had to be explained to Occupational Therapists using both written and spoken language. The strength of the present work is the validation of CP ICF Core Set following its development by Schiariti (45).

The current study was limited in that the results may not be generalizable to the whole world occupational therapists. Before the study, we considered one month for each Delphi round, but in practice, each round lasted about two months. Criteria considered for agreement in this study was 75% but in some studies cut-off 50% was considered (41). Considering higher percentage in this study resulted in some phases of the study took time longer than intended. Further international studies are needed so that cultural effects can be taken into account. It is suggested to validate of ICF Core Set for children 6 to 18 yr with cerebral palsy. 

**In conclusion, **about 60% of the code-categories of the comprehensive version of ICF Core Set of CP were confirmed by Occupational Therapists with agreement greater than 75%. This shows the high validity of this version of ICF Core Set from the perspective of the Iranian Occupational Therapists. The final version considering all components of health, activity, and participation. The Occupational Therapists perspective is well reflected in it. Iranian Occupational Therapists can use this framework as a starting point in the assessment of children aged 0–6 yr with CP, not as an assessment tool. This kind of assessment based on the ICF, not only satisfies the clients but also ensures that intervention results can be effectively monitored. The framework ensures that all dimensions of functioning and disability are covered according to principles of Occupational Therapy. 
